# IR-Spectroscopic Study of Complex Formation of Nitrogen Oxides (NO, N_2_O) with Cationic Forms of Zeolites and the Reactivity of Adsorbed Species in CO and CH_4_ Oxidation

**DOI:** 10.3390/molecules27010055

**Published:** 2021-12-22

**Authors:** Alexander L. Kustov, Leonid M. Kustov

**Affiliations:** 1 Chemistry Department, Moscow State University, 1 Leninskie Gory, Bldg. 3, 119991 Moscow, Russia; kyst@list.ru; 2 N.D. Zelinsky Institute of Organic Chemistry RAS, 47 Leninsky Prosp., 119991 Moscow, Russia; 3 Institute of Ecotechnologies and Engineering, National University of Science and Technology MISiS, 4 Leninsky Prosp., 119049 Moscow, Russia

**Keywords:** zeolite, N_2_O, NO, cations, CO oxidation, CH_4_ oxidation, diffuse reflectance IR spectroscopy

## Abstract

The formation of complexes and disproportionation of nitrogen oxides (NO, N_2_O) on cationic forms of LTA, FAU, and MOR zeolites was investigated by diffuse-reflectance IR spectroscopy. N_2_O is adsorbed on the samples under study in the molecular form and the frequencies of the first overtone of the stretching vibrations ν^1^_0–2_ and the combination bands of the stretching vibrations with other vibrational modes for N_2_O complexes with cationic sites in zeolites (ν^3^_0–1_ + ν^1^_0–1_, ν^1^_0–1_ + δ_0–2_) are more significantly influenced by the nature of the zeolite. The presence of several IR bands in the region of 2400–2600 cm^−1^ (the ν^1^_0–1_ + δ_0–2_ transitions) for different zeolite types was explained by the availability of different localization sites for cations in these zeolites. The frequencies in this region also depend on the nature of the cation (its charge and radius). The data can be explained by the specific geometry of the N_2_O complex formed, presumably two-point adsorption of N_2_O on a cation and a neighboring oxygen atom of the framework. Adsorption of CO or CH_4_ on the samples with preliminarily adsorbed N_2_O at 20–180 °C does not result in any oxidation of these molecules. NO^+^ and N_2_O_3_ species formed by disproportionation of NO are capable of oxidizing CO and CH_4_ molecules to CO_2_, whereas NO_x_ is reduced simultaneously to N_2_ or N_2_O. The peculiarities in the behavior of cationic forms of different zeolites with respect to adsorbed nitrogen oxides determined by different density and localization of cations have been established.

## 1. Introduction

One of the major problems of environmental protection is the problem of purification of exhaust gases of chemical and coke producing industry as well as power plants from nitrogen oxides [1]. Zeolite catalysts are considered among the prospective alternatives to the existing honeycomb ceramic catalysts for selective reduction of NO_x_ to N_2_ by NH_3_. Other reducing agents such as CO that are present in considerable amounts in exhaust gases together with NO_x_ and CH_4_ can also be used for NO_x_ abatement [2,3].

It is known that nitrogen oxides are often used as spectroscopic probe molecules for studying surface sites of oxide and zeolite adsorbents and catalysts by the methods of IR and ESR spectroscopy [4,5]. Cationic forms of zeolites are the example of the systems that are characterized by the formation of a variety of adsorbed species produced upon adsorption of NO_x_. These species are formed as a result of either complex formation with surface sites or via chemical reactions occurring in the course of adsorption [6,7]. The transformation of nitrogen oxides accompanying their adsorption have been observed for zeolites containing not only transition metal ions [8], for instance copper, which are used in selective catalytic reduction of NO_x_, and reduced metals [9], but also for non-transition metal ions [10].

The recent review by Huang et al. [11] summarizes the recent research related to NO_x_ adsorption on zeolites in comparison with carbon adsorbents and metal–organic frameworks (MOFs). A conclusion was drawn about the stronger adsorption of NO_x_ on zeolites compared with other adsorbents.

Most studies on NO_x_ adsorption and removal from flue gases using zeolites as adsorbents and catalysts are devoted to Cu- and Fe-containing systems [12,13,14,15]. Sun et al. studied a series of micro- and meso-porous MOFs and zeolites for the removal of SO_2_ and NO_x_ from flue gases [12]. For the comparison, 12 porous materials were taken as adsorbents for removing NO_x_ from flue gases, including four all-silica zeolites, six MOFs, and two zeolitic imidazolate frameworks (ZIFs). The data obtained demonstrate that Cu-BTC is characterized by the highest adsorption of NO_x_, and the other adsorbents could not ensure effective purification. Skarlis et al. [13] studied the capture of NO_x_ and NO oxidation by the Fe-BEA zeolite using FTIR spectroscopy. Liu et al. [14] suggested the high-silica zeolite Cu-SSZ-13 for efficient NO_x_ removal by adsorption. Pan et al. [15] studied NO_x_ adsorption on different Fe/zeolites in the presence of interfering sour gases, such as SO_2_, CO_2_ as well as water and found the following order of the decreasing NO_x_ adsorption capacity of the Fe/zeolites: Fe/MOR > Fe/FER > Fe/ZSM-5 > Fe/Beta. However, the adsorption capacity of Fe/MOR for NO_x_ did not exceed 3.2 mg g^–1^. The activity of adsorbed forms of NO_x_ in reduction with C_3_H_6_ was established.

The NO decomposition into nitrogen and oxygen was shown to be catalyzed by copper- and iron-exchanged ZSM-5 zeolites under conditions of microwave heating [16]. Because of the rapid heating, NO is decomposed without desorption of NO. The presence of oxygen and water vapor in NO almost does not affect the adsorption properties of the zeolites with respect to NO.

The discussed publications are lacking for the spectroscopic identification of adsorbed species arising on cation-exchanged zeolites after adsorption of NO and other NO_x_ molecules. A few papers, however, report on the formation of some species that are supposed to be active in oxidation of the reducing agents used for removal of NO_x_, including NH_3_, CO, and hydrocarbons. FTIR spectroscopy was used to study the Cu and Fe exchanged zeolites in the selective catalytic reduction of nitrogen oxides (NO_x_) with NH_3_ at 200 °C [17]. Noteworthy that the surface NO_x_ adsorption complexes observed on these two catalysts were different. IR bands assigned to surface nitrate/nitrite groups were found for the Cu catalyst, whereas for the Fe catalyst, IR bands of surface nitro groups were also observed, in addition to nitrate/nitrite species. The nitrate/nitrite and nitro groups revealed high activity toward NH_3_. The higher activity on the Fe catalyst at the optimum NO:NO_2_ = 1:1 ratio was explained by the high intrinsic activity of the nitro groups formed on this catalyst.

Decomposition of NO has also been studied on Cu,La-ZSM-5 zeolite in the temperature range from 50 to 520 °C [18]. The mechanism of N_2_O decomposition on binuclear FeII(μ-O)(μ-OH)FeII iron sites in Fe-ZSM-5 zeolite was investigated by the DFT method [19]. The dissociation of N_2_O is found to be highly energetic but the energy barrier associated with the atomic oxygen migration is higher. Selective catalytic reduction of NO_2_ to N_2_ with ammonia at 298 K was studied on NaY and CuY zeolites by in situ Fourier transform infrared spectroscopy [20]. Silanol groups and extra-framework aluminum sites provide adsorption sites for NO_2_ and NH_3_.

Earlier publications [21,22,23] also demonstrated the ability of non-transition metal ions to activate reactions of NO on cationic forms of ZSM-5 and SAPO-34 zeolites, including NO disproportionation. The spectra provided evidence for the formation of nitrous oxide and nitrate species. N_2_O and N_2_O_3_ were found as major products.

NO adsorbed at 77 K on the titanosilicate molecular sieve ETS-10 [24] forms M^+^···(NO) adducts, M^+^=Na, K exhibiting IR absorption bands in the range 1820–1900 cm^−1^. Due to NO to dimerization, cis- and trans-dimers interacting with alkali cations could be observed.

Adsorption of NO and NO_2_ on Ba-, Cs-, Na-, and Li-Y zeolites at 373–833 K revealed the following trend in the adsorption capacity that decreases in the order: Ba-Y > Cs-Y > Na-Y > Li-Y. [25].

NO adsorption on CoY zeolite in comparison with Co-ZSM-5 zeolite was also studied by IR spectroscopy [26]. Dinitrosyl species (absorption bands at 1900 and 1819 cm^−1^) which are rather stable are not involved in the SCR process. Monodentate nitrates formed on Co-ZSM-5 (1540 cm^−1^) easily interact with hydrocarbons and are active in SCR. No monodentate nitrates are formed on CoY. Stable symmetric nitrates (1488 and 1483 cm^−1^) and less stable species, probably bidentate nitrates (1620 and 1320 cm^−1^), appear instead. The symmetric nitrates are converted, during evacuation, into nitro compounds (1563 and 1383 cm^−1^) that are not removed by evacuation at 743 K. Interaction of methane with nitrates on CoY leads to their partial reduction to nitro compounds.

The reaction of NO disproportionation occurs also on Na-chabasite, Na-faujasite, as well as on zeolites CaY as follows [10,27]: 4NO = N_2_O + N_2_O_3_.

The active sites of this reaction are supposed to be metal cations. However, the mechanism of this process and the effect of the zeolite structure and composition on the reaction course are virtually unstudied. The peculiarities of the processes of CO and CH_4_ oxidation by adsorbed NO were also not quite well investigated.

On the contrary to NO adsorption, adsorption of N_2_O has been studied by infrared spectroscopy to a lesser extent [28,29,30]. Two bands at 2238 and 2008 cm^−1^ were attributed to adsorbed N_2_O on Ir/oxide catalysts. Ir/ZrO_2_ and Ir/Al_2_O_3_, which displayed high activities in N_2_O decomposition, had significant band intensities attributed to N_2_O. The authors [28] suggested a correlation between the N_2_O adsorption and the catalytic activity in N_2_O decomposition.

Adsorption of N_2_O on reduced ceria was shown [31] to proceed with dissociation leading to the formation of N_2_ with simultaneous filling the oxygen vacancies and oxidation of Ce^3+^.

The goal of this work was to study the formation of complexes and the transformations of NO and N_2_O, including disproportionation and oxidation of CO and CH_4_ on cationic forms (alkaline and alkali-earth) of zeolites differing in the stricture and composition.

## 2. Results and Discussion

### 2.1. N_2_O Adsorption on Cationic Forms of Zeolites

**Figure 1:**
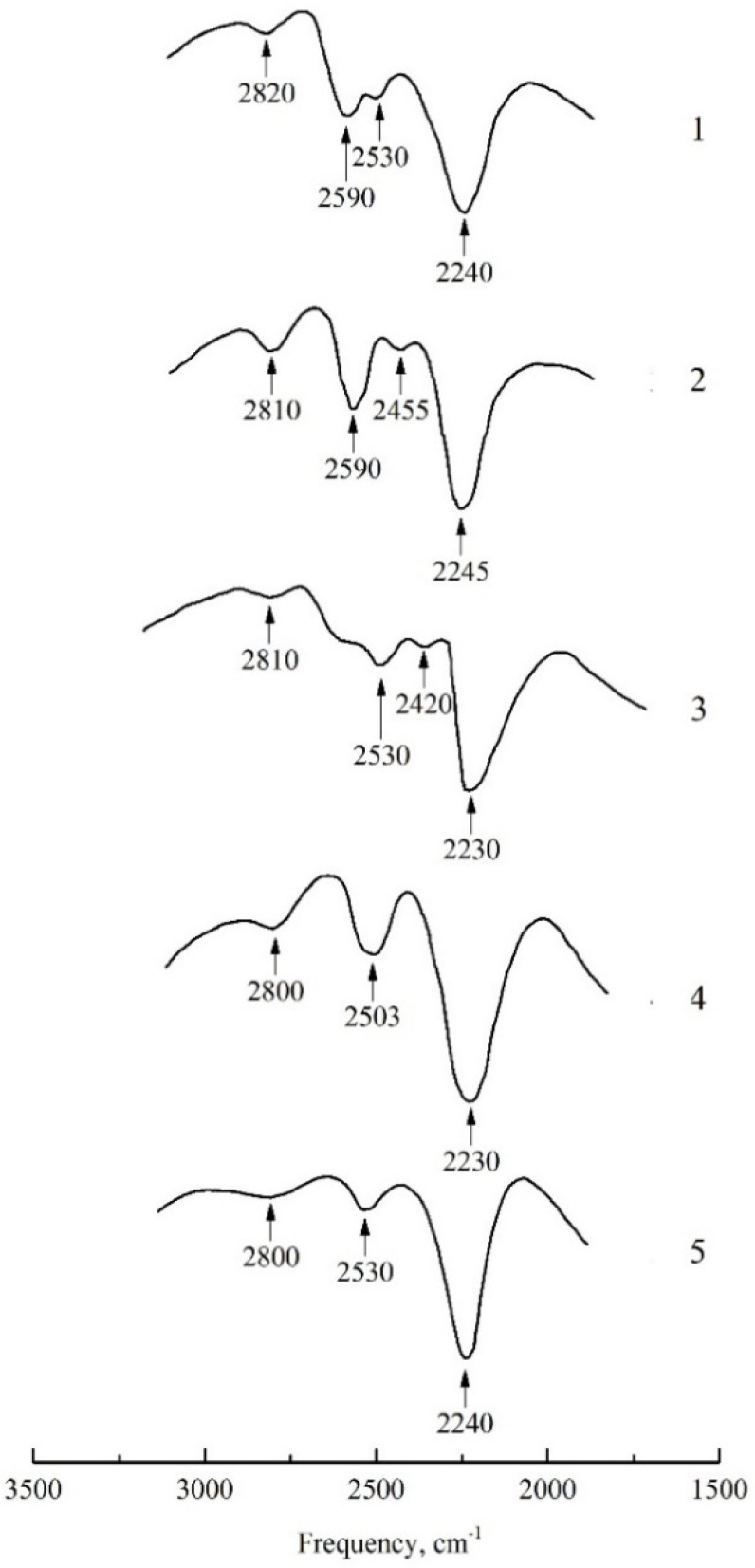
IR spectra of N_2_O adsorbed at 20 °C and 10 Torr on Na-MOR (1), Na-LTA (2), Na-FAU (Y) (3), Ca-FAU (Y) (4), and Mg-MOR (5).

Figure 1 presents IR spectra measured after N_2_O adsorption on Na-MOR (1), Na-FAU (2), Na-LTA (3), Ca-FAU (4), and Mg-MOR (5) zeolites. Adsorption of N_2_O on these samples results in the appearance of a set of bands in IR spectra attributed to the main transition of the ν^3^ stretching vibrations and combination bands and overtones of adsorbed N_2_O molecules. By analogy to the interpretation of the IR spectrum of gaseous N_2_O [32] and in agreement with the assignment made in our previous publication [33], the most intense bands in the region of 2230–2245 cm^−1^ can be ascribed to the ν^3^ stretching vibrations of N_2_O. The bands in the region of 2420–2510 cm^−1^ are assigned to the combination of the ν^1^ stretching and the first overtone of the bending vibrations (ν^1^_0–1_ + δ_0–2_), whereas the bands in the range of 2590–2600 cm^−1^ can be attributed to the first overtone of the ν^1^ stretching vibrations (ν^1^_0–2_) of the N_2_O molecule. The bands with the maxima at 2800–2820 belong to the combination of the ν^3^ stretching vibrations and the main tone of the bending vibrations (ν^3^_0–1_ + δ_0–1_). Moreover, weak bands in the region of 3475–3530 cm^−1^ have been identified (not shown in the figure) and assigned to the combination band of the ν^3^ stretching and ν^1^ stretching vibrations (ν^3^_0–1_ + ν^1^_0–1_).

Table 1 presents the frequencies of IR bands of N_2_O adsorbed on cations in the zeolites under study, as well as the corresponding frequencies of N_2_O in the gas phase. It is seen from the data of Table 1 that N_2_O is adsorbed on the samples under study in the molecular form. Comparison of the spectra in Figure 1 and data of Table 1 shows that the maximum of the band of ν^3^ stretching vibrations of N_2_O remains virtually unchanged for different zeolites and different cations (Na, Ca, Mg) and is equal to 2230–2245 cm^−1^. The maxima of the bands assigned to the combination ν^3^_0–1_ + δ_0–1_ is also not sensitive to the nature of the zeolite and cation in the samples under study and are ranged within 2800–2820 cm^−1^.

The frequencies of the first overtone of the ν^1^ stretching vibrations ν^1^_0–2_ and the combination bands of the ν^1^ stretching vibrations with other vibrational modes for N_2_O complexes with cationic sites in zeolites (ν^3^_0–1_ + ν^1^_0–1_, ν^1^_0–1_ + δ_0–2_) are more significantly influenced by the nature of the zeolite. Obviously, the cations in zeolites are responsible for the formation of complexes with N_2_O. In such a case, one may assume that the differences in the spectra and band positions as well as the number of bands in the region of 2400–2600 cm^−1^ (the ν^1^_0–1_ + δ_0–2_ transitions) for different zeolite types may be explained by the presence of several possible localization sites for cations in these zeolites, i.e., by the set of different adsorption complexes of N_2_O with cations located in different sites in the zeolite framework. The zeolite NaA is characterized by localization of sodium cations in the centers of six-membered oxygen rings at the faces of the cuboctahedron (8 sites out of 12 possible sites in this zeolite). Accordingly, the IR spectrum of N_2_O adsorbed on Na-LTA zeolite demonstrates a single band in the region of ν^1^_0–1_ + δ_0–2_ at 2455 cm^−1^, and only one overtone band at 2590 cm^−1^ is observed in the region of the ν^1^_0–2_ transitions. For NaY (Na-FAU) and Na-MOR zeolites, the number of sites populated with cations increases to two–3three, for instance, S_I_, S_I_^’^, and S_II_ for NaY and the sites in the main channels, and in the side channels of mordenite. In agreement with the known distribution of sodium cations in these zeolites, the IR spectra of N_2_O adsorbed on these zeolites demonstrate a more complicated pattern with a superposition of several absorption bands in the region of ν^1^_0–1_ + δ_0–2_ and ν^1^_0–2_ transitions at 2400–2600 cm^−1^.

Evidently, the observed spectra in the region of ν^1^_0–1_ + δ_0–2_ and ν^1^_0–2_ transitions at 2400–2600 cm^−1^ depend not only on the structure type of the zeolite, but also on the nature of the cation (its charge and radius). Indeed, when passing from the Na-form of zeolite Y and mordenite (spectra 1 and 3 in Figure 1) to the alkaline-earth cationic forms (spectra 4, 5 in Figure 1, respectively), the spectra in the region of 2400–2600 cm^−1^ become not so complicated, which may be accounted for by the more uniform distribution of alkaline-earth cations in these zeolites compared with the alkaline cations, in agreement with the literature data [34].

Thus, adsorption of N_2_O on alkaline and alkaline-earth cationic forms of zeolites results in more significant changes of the frequencies of the ν^1^ stretching vibrations, but not ν^3^ stretching vibrations. This result, most likely, can be explained by the specific geometry of the complex formed, presumably two-point adsorption of N_2_O with the oxygen atom of the N_2_O molecule being coordinated by a cation and one of the nitrogen atoms (presumably, the central atom) being linked to a neighboring oxygen atom of the framework, as well as by the lower characteristics of the ν^1^ stretching vibrations compared with the ν^3^ stretching vibrations.

**Scheme 1:**
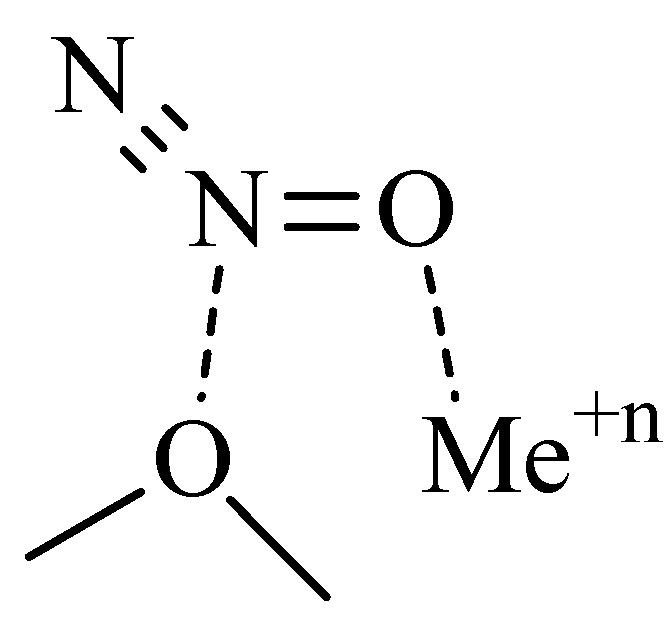
N_2_O complex with a cation and neighboring oxygen anion of the framework in zeolites.

The analysis of the spectroscopic data with the earlier obtained results [33] shows that the IR spectra of N_2_O adsorbed on metal cations are qualitatively similar to those observed for the N_2_O complexes on Lewis acid sites of zeolites. According to quantum-chemical calculations [33], the N_2_O molecule is adsorbed by a two-point mechanism involving not only the low-coordinated ion of the Lewis acid site but also the neighboring oxygen atom of the zeolite framework possessing basic properties (Scheme 1). In such a case, the geometry of the N_2_O molecule changes significantly, in particular the ONN angle and the charge distribution in the N_2_O molecule. Such adsorption complexes with a two-point geometry, according to quantum chemical calculations, are characterized by the more favorable energy of the interaction (E = 62 kcal/mol) compared with one-point interaction in the case of N_2_O complexes with proton sites (E = 16 kcal/mol), in agreement with the data on the strength of N_2_O adsorption on these sites.

It has been shown in [33] that complexes of N_2_O with OH groups are destroyed even by a short evacuation at room temperature, whereas the complexes with strong Lewis acid sites are stable until 180–230 °C and are decomposed with evolution of N_2_ into the gas phase and chemisorption of atomic oxygen. This atomically chemisorbed oxygen exhibits pronounced oxidation properties towards different molecules, such as H_2_, CO, and CH_4_, etc. The complexes of N_2_O with metal cations in cationic forms of zeolites are likely characterized by a similar two-point geometry by analogy to the interaction with Lewis acid sites.

At the same time, the strength of N_2_O adsorption on cationic forms, though being higher compared with complexes with OH groups, is nevertheless lower than that for the complexes with strong Lewis acid sites of zeolites. Furthermore, in the case of the cationic forms of zeolites, further adsorption of CO or CH_4_ on the samples with preliminarily adsorbed N_2_O at 20–180 °C does not result in any oxidation of such molecules. Therefore, the increase in the temperature to 180–230 °C does not lead to the N_2_O decomposition on cationic forms of alkaline and alkaline-earth forms of zeolites as well as to the formation of chemisorbed atomic oxygen species and thereby to the appearance of any oxidation properties related to the chemisorption of oxygen. This reflects the principal difference in the behavior of alkaline and alkaline-earth cations compared with strong Lewis acid sites of zeolites.

### 2.2. Adsorption of NO on Y Type Zeolites

**Figure 2:**
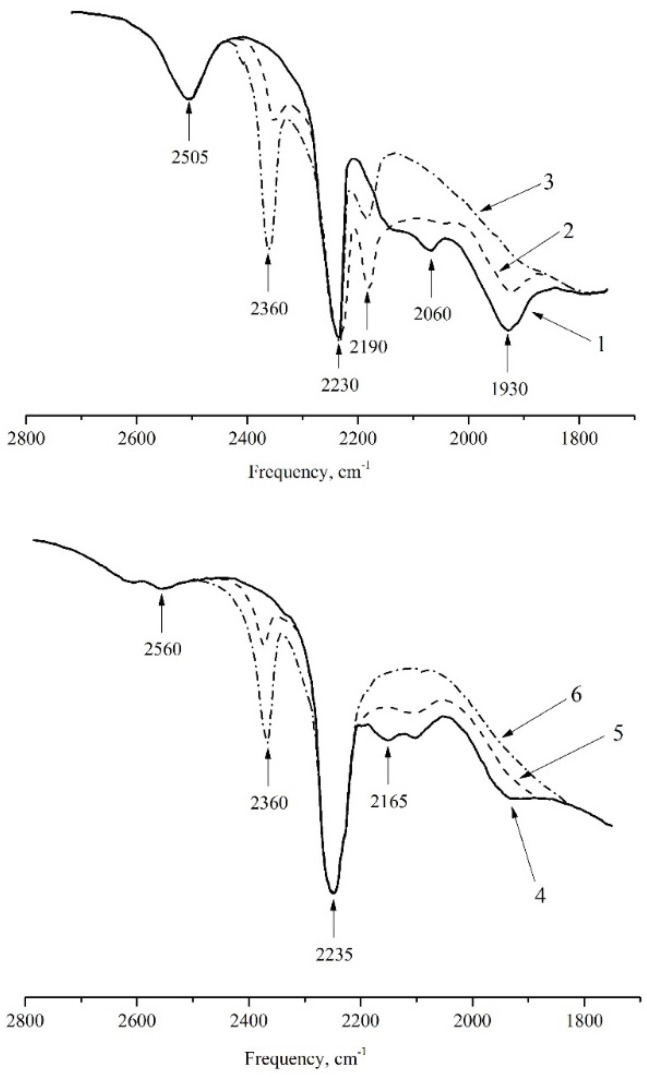
IR spectra of NO adsorbed at 20 °C and 10 Torr on Ca-FAU (Y) and Na-FAU (Y) (1, 4, respectively) followed by adsorption of CO at 20 °C and 10 Torr CO (2, 5, respectively), and heating at 150 °C for 1 h (3, 6, respectively).

Figure 2 presents IR spectra measured after adsorption of NO at 20 °C on NaY and Ca-Y zeolites. These spectra are similar to the earlier observed spectra of NO adsorbed on Na- and Ca-forms of different zeolites [6,35].

Unlike N_2_O, adsorption of NO on alkaline and alkaline-earth forms of zeolites does not produce molecular forms of adsorbed NO, as far as the spectra exhibit no absorption bands in the region of stretching vibrations of NO close to the frequency for the gas phase NO at 1880–1890 cm^−1^, which can be shifted to 1860–1870 cm^−1^ in the case of NO adsorption on some transition metal ions [24,26]. The absence of the bands assigned to molecular forms of NO is explained by the formation of charged forms of NO and by NO disproportionation in the course of adsorption with the formation of complexes containing NO_x_ with nitrogen in different oxidation states.

**Scheme 2:**
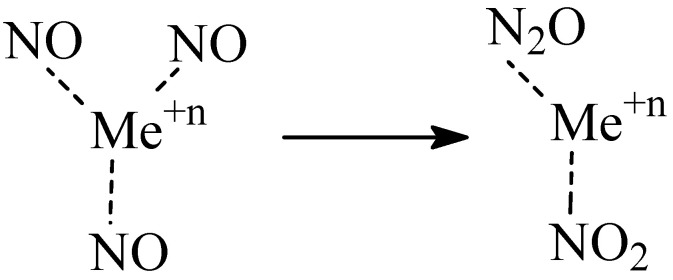
NO complexes and their transformations on cationic forms of zeolites.

On the basis of the literature data [6], the absorption bands observed in IR spectra can be attributed to partially charged NO^+δ^ or NO^+^ species and disproportionation products in the following way. The bands at 2505 and 2230 cm^−1^ are assigned to N_2_O molecules, the bands at 2150–2080 cm^−1^—to charged NO^+δ^ or NO^+^ species, whereas the band at 1930 cm^−1^ can be ascribed to NO_2_ or N_2_O_3_ (or an NO-NO_2_ dimeric complex) (Scheme 2). Obviously, other forms can be produced upon disproportionation (NO_2_, NO_3_^−^, etc.), which are not visualized in diffuse reflectance IR spectra in the low-frequency range of the spectra, but the bands of such species were observed earlier in transmittance IR spectra [6].

The further adsorption of CO at 20 °C on the samples of NaY and CaY zeolites with preadsorbed NO results in CO oxidation with the formation of CO_2_ identified in IR spectra by the band at 2360 cm^−1^. During this experiment, the bands assigned to N_2_O (2505, 2230–2250 cm^−1^) remain unchanged in their intensity. On the contrary, the intensities of the bands attributed to NO^+δ^ (2060 cm^−1^) and especially N_2_O_3_ species (1930 cm^−1^) decrease significantly. After heating the samples at 150 °C for 30 min, the bands of NO^+δ^ and N_2_O_3_ completely disappear, while the intensity of the CO_2_ band at 2360 cm^−1^ reaches the maximum intensity (Figure 2, spectrum 1). The reaction on NaY proceeds in a similar way (Figure 2, spectrum 4).

**Figure 3:**
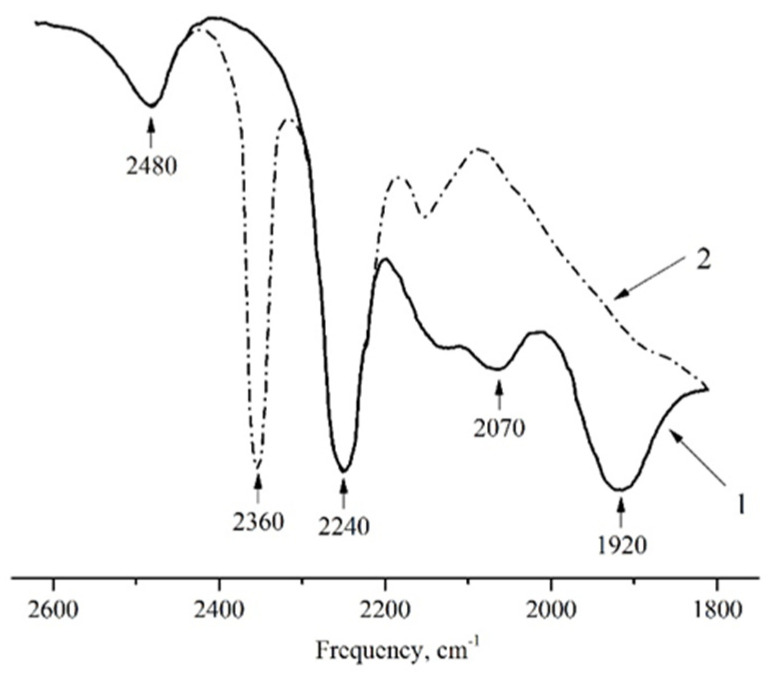
IR spectra of NO adsorbed at 20 °C and 10 Torr on Ca-FAU (Y) zeolite (1) followed by adsorption of CH_4_ (20 °C, 10 Torr) (2).

Methane adsorption at room temperature on the NaY and CaY zeolites with preadsorbed NO also leads to the formation of CO_2_, especially after heating the samples at 100–200 °C (Figure 3).

**Figure 4:**
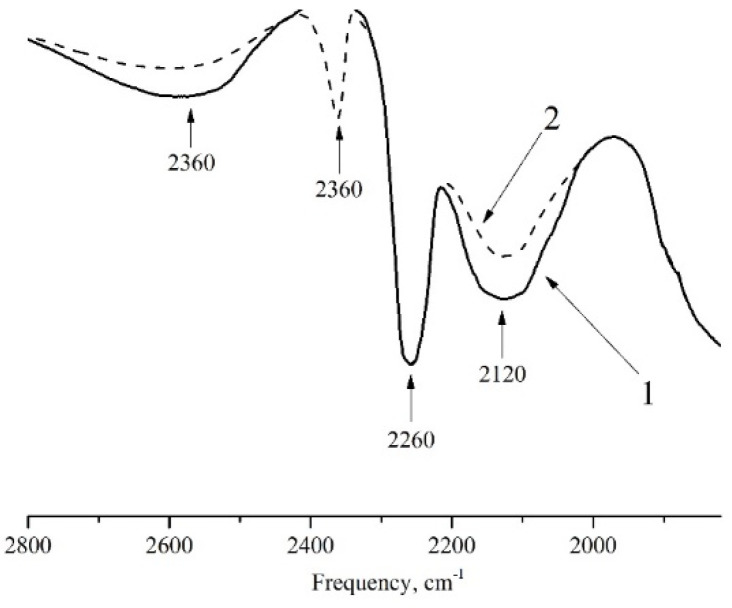
IR spectra of NO adsorbed at 20 °C and 10 Torr on Ca-MOR zeolite (1) followed by adsorption of CO (20 °C, 10 Torr) and heating at 250 °C (2).

Unlike the studied NaY and CaY zeolites, the reaction of NO disproportionation on cationic forms of mordenite, including Na-MOR, Ca-MOR, Mg-MOR, and Sr-MOR proceeds in a different manner. Figure 4 displays IR spectra measured after NO adsorption on Ca-MOR. In this case (Figure 4, spectrum 1), only the bands assigned to N_2_O (2260 cm^−1^) and NO^+δ^ (2120 cm^−1^) are observed in the spectra. No forms of adsorbed NO that can be identified as N_2_O_3_ (the possible band at 1930 cm^−1^) are seen in the spectra of mordenites. The further admission of CO on the samples with preadsorbed NO at 20 °C does not result in CO oxidation to CO_2_. Heating at 150 °C for 30 min does not change the spectral pattern, i.e., no signs of CO_2_ formation were revealed. Only after heating at 300 °C for 1 h, the spectrum demonstrates weak-intensity absorption bands assigned to CO_2_ (Figure 4, spectrum 2).

The reaction of methane oxidation with NO on Ca-mordenite also occurs only at elevated temperatures over 300 °C and results in CO_2_ formation. The Mg-MOR, Sr-MOR, and Na-MOR zeolites demonstrate about the same pattern in the reactions NO + CO and NO + CH_4_.

## 3. Materials and Methods

Na-, Ca-, and Mg-forms of LTA (Si/Al = 1.0), FAU (Si/Al = 2.35), and MOR (Si/Al = 5.0) zeolites were studied in this work. The extents of ion exchange of Na for Ca or Mg were 80–90%. The ion exchange was carried out at 20 °C using aqueous solutions of Ca and Mg nitrates. The samples were washed with distilled water and dried at 150 °C. The samples were placed in quartz IR ampules supplied with a side finger with a CaF_2_ window for the measurements of diffuse reflectance IR spectra. The finger was loaded with a CaF_2_ powder (0.1–0.2 mm), which was pretreated at 150 °C for 0.5 h. The powdered zeolite samples (0.1–0.2 mm) were pretreated in the quartz part of the cell in a vacuum at 450–700 °C for 4 h. The rate of the temperature increase was 4 °C/min. Then the samples were mixed with the pretreated CaF_2_ powder under vacuum in the zeolite: CaF_2_ weight ratio of 1:5 in order to obtain high-quality diffuse reflectance IR spectra in the region of 2300–1600 cm^−^^1^. Diffuse reflectance IR spectra were measured using a PerkinElmer 580B spectrometer in the frequency range of 1600–4000 cm^−1^. The range below 1600 cm^−1^ overlapped with absorption of quartz and CaF_2_ and therefore was not studied. The intensity of the bands in the DRIFT spectra was expressed in Kubelka–Munk units. The preparation and purification of NO are described in [36]. Nitrous oxide (N_2_O), methane, and carbon monoxide were adsorbed at 20 °C and a pressure of 1–100 Torr. Nitrogen monoxide was adsorbed on the samples at −196 °C or at 20 °C at a pressure of 1–50 Torr.

## 4. Conclusions

N_2_O was shown to be adsorbed on the cationic forms of zeolites in the molecular form with the frequencies of the first overtone of the stretching vibrations ν^1^_0–2_ and the combination bands of the stretching vibrations with other vibrational modes for N_2_O complexes with cationic sites in zeolites (ν^3^_0–1_ + ν^1^_0–1_, ν^1^_0–1_ + δ_0–2_) being strongly influenced by the nature of the zeolite (the number of localization sites and the nature of the cation). The data obtained can be explained by the specific geometry of the N_2_O complex formed, presumably with the oxygen atom of the N_2_O molecule being coordinated by a cation and one of the nitrogen atoms (presumably, the central atom) being linked to a neighboring oxygen atom of the framework. CO or CH_4_ cannot be oxidized on the samples with preliminarily adsorbed N_2_O at 20–180 °C.

The reaction of NO disproportionation on different types of zeolites proceeds in different ways. This may be explained by the different density of cations (distribution) and by the different porous structure of the zeolites. For zeolites Y with the FAU structure, the density of cations is high, whereas their porous structure determined by the presence of large and small cavities is beneficial for the localization of cations in close vicinity to each other. These factors increase the probability of the occurrence of bimolecular processes in the coordination sphere of the closely located cation pairs. As it was shown, N_2_O_3_ species are formed in a significant concentration in such a case, which are evidently most active in the reactions of CO and CH_4_ oxidation to CO_2_.

On the contrary, in the case of mordenites, which are characterized by the low density of exchanged cations and the channel structure is beneficial for the increase in the distance between neighboring cations, the possibility of N_2_O_3_ formation is much lower compared with faujasites. As a result, the activity of the cationic forms of mordenite in the reactions of CO and CH_4_ oxidation decreases significantly.

## Figures and Tables

**Table 1:** Frequencies (cm^−1^) of IR bands of N_2_O adsorbed on cationic forms of the zeolites under study.

Spectral Transition	N_2_O in the Gas Phase	Ca-FAU	Na-LTA	Na-FAU	Na-MOR	Mg-MOR
ν^3^_0–1_	2224	2230	2245	2230	2240	2240
ν^1^_0–1_	1285	-	-	-	-	-
δ_0–1_	589	-	-	-	-	-
δ_0–2_	1169	-	-	-	-	-
ν^1^_0–1_ + δ_0–2_	2460	-	2455	2420	-	-
ν^1^_0–2_	2562	2503	2590	2600	2530, 2590	2530, 2600
ν^3^_0–1_ + δ_0–1_	2796	2800	2810	2810	2820	2800
ν^3^_0–1_ + δ_0–2_	3360	-	-	-	-	-
ν^3^_0–1_ + ν^1^_0–1_	3489	3475	-	3475, 3530	3440, 3540	3480

## Data Availability

The data presented in this study are not available from the authors.

## Sample Availability

Samples of the compounds are not available from the authors.
